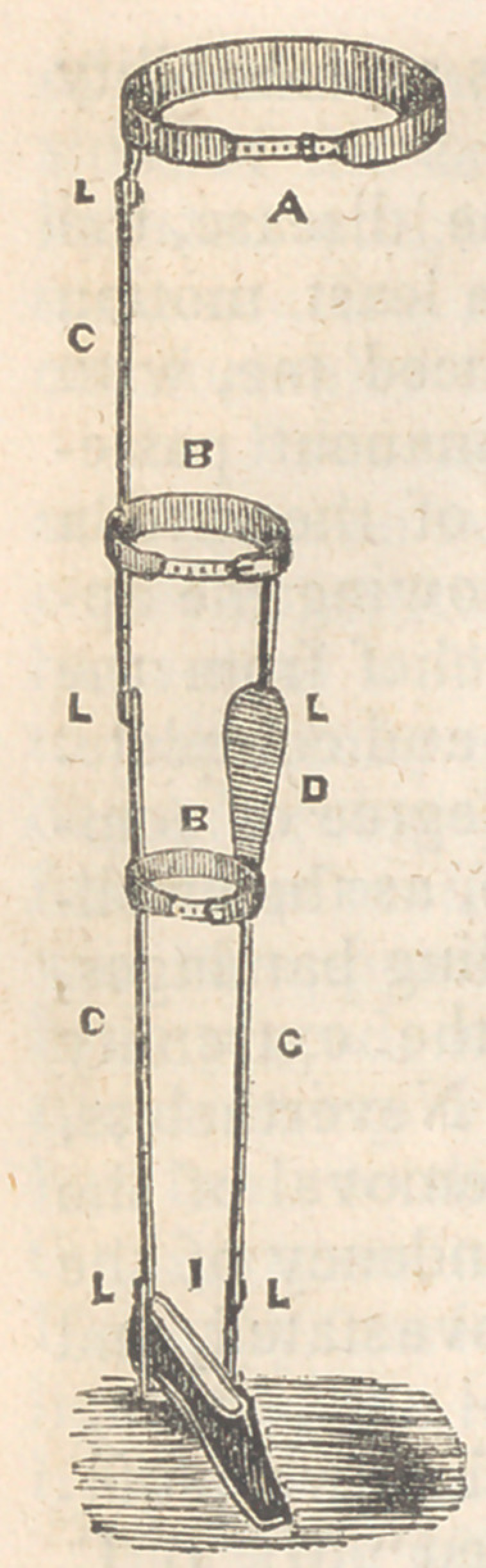# False Anchylosis of the Knee Joint, Treated with Steel Springs, &c., &c.

**Published:** 1847-07

**Authors:** James Bryan

**Affiliations:** Lecturer on Surgery — formerly Professor of Surgery and Medical Jurisprudence in the Academy of Medicine, Vermont


*False Anchylosis of the Knee Joint, treated with Steel Springs, &c., &c.* By JAMES BRYAN, M. D., Lecturer on Surgery—formerly Professor of Surgery and Medical Jurisprudence in the Academy of Medicine, Vermont.

Mrs. L. aged about 35 years, had been delivered of a (first) healthy child by means of instruments but a few days, when she was attacked with great pain and swelling of the right leg and thigh, exhibiting all the symptoms of *phlegmasia alba dolens.* My friend Dr. Joseph Warrington, who was the attending accoucheur, requested me to see her. The swelling, pain, and heat, were very great, and terminated after a protracted course of treatment, during which my friend Dr. Rhea Barton saw the case with me, in incomplete anchylosis of the knee-joint, with great contraction of the flexor muscles of the limb. The heel was drawn up and it was impossible for the patient to bring it to the ground; very little weight could be borne on the toes and ball of the foot, the only parts permitted to touch the floor.

The contraction of the muscles and consequent angularity of the leg increased daily, so much so that there was a probability of a total loss of its use.

During the treatment of the acute stage of the disease, the severity of the pain, amounting to agony, on the least motion taking place in the excessively tumified limb, induced me, with the assistance of Dr. A. M. Pena, to apply a permanent pasteboard case to the whole limb, leaving the region of the patella only uncovered. This was for the purpose of allowing the application of remedies to the diseased joint. The relief from the general support given by the case, was immediate and complete. She was enabled to rest and to sleep with some degree of comfort. The case was kept in contact with the limb, as the swelling subsided, by gradually tightening the surrounding bandages, and in this way, the patient lying on her back, the extremity was retained in a position not far from rectilinear. Nevertheless, after the subsidence of the inflammation and the removal of the case, as soon as the muscles began to act, the ascendency of the flexors was so great, that the deformity was, as above stated, and continued daily to increase.

At this time, when the general health of the patient was sufficiently restored to require air and exercise, I directed "Mr. B. C. Everett, Principal of the Surgeon's Bandage Institute," to manufacture a steel support for the limb, which would have for its object to throw the greater part of the weight of the body (through the spring) from the foot to the pelvis—or rather from the pelvis to the foot, thus leaving it optional with the patient how much weight she should place upon the foot. It was also to give lateral support to the joints, particularly the knee joint, so that the patient might attempt the use of the leg without bearing much weight upon it, at the same time, by the lateral pressure, the parts should be so supported as to secure them from distortion in any direction. The patient was directed to use frictions with dry salt, salt and water, alternating with animal oils and liniments, such as lard, volatile soap, and camphorated liniments, &c., &c. The limb was to be used gently but firmly and perseveringly, treading of course on the toes and ball of the foot. I strictly forbade her wearing a high-heeled shoe or boot, hoping by the above means to bring down the heel and straighten the knee joint.

At the end of some six months, the strength of the limb had so increased that she could walk upon it with little difficulty, and before a year had passed the instrument was taken off, and her locomotion restored nearly to the natural manner, only a slight shortening of the limb remaining, producing in her walk an almost imperceptible halting. This condition remains to the present time, now almost four years. She is a lady of great activity, and daily takes a large amount of exercise on foot.

The accompanying wood cut represents the instrument used. A, is a circular spring well padded, to pass around the pelvis and fasten in front by means of a strap and buckle. L L L, the joints corresponding to those of the hip, knee and ankle, C C, the thigh and leg pieces. B B, metallic bands and straps for the thigh and leg. I, the shoe and cross piece.

Since writing the above, I have observed in the fourteenth part of "Braithwaite's Retrospect" for 1847, an article by Anthony Colling Brownless, Esq., of London, "On the value of position and mechanical support in the treatment of Diseased Joints, with special reference to the knee-joint."

"The subsequent usefulness of diseased joints," remarks the editor of the above very excellent publication, "depends, 1st, upon the position they are allowed to assume during the active stage; and 2dly, upon proper support in the convalescent stage, when the activity of the disease being subdued, the patient is beginning to use the limb." In reference to the support of the limb, during the active stage of the disease, the following paragraph from Dr. Brownless' paper accords with the experience of surgeons.

"During the active progress of the disease, any splint or apparatus, which will at once maintain the joint in a desirable position, prevent any considerable motion, and be comfortable to the patient, will fulfil all our intentions. Perhaps the strong pasteboard or undressed leather splint,* adapted to the part whilst wet, and afterwards softly padded with lint, or, what is better, goldsmith's or jeweller's wool, will answer as well as anything else, it being light, and at the same time giving good support; but whatever the apparatus may be, no pains should be spared in fitting it in such a manner to the part, as to be perfectly easy to the patient, at the same time that it gives steadiness to the limb, by extending sufficiently above and below the joint.

The limb should lay in a sort of case, which should be long enough to receive the calf of the leg, and also extend well up the thigh." We concur also in the following:

"By the use, then, of this plan to diseased joints, we obtain more or less the following important ends:—first, the alleviation of the sufferings of the patient; secondly, the lessening the liability to repeated attacks of inflammation, and, consequently,

* The leather case is also recommended by Sir B. Brodie. See Diseases of Joints.

thirdly, the acceleration of the cure ; fourthly, the prevention of deformity, if the disease terminates in anchlyosis, partial or complete; and, fifthly, the ultimate utility of the limb."

But in the "after treatment" my "notions" and apparatus differ very materially from his—after stating the necessity of support to joints thus situated, during the convalescent stage, he proceeds to say :—" I know of no better support for a knee-joint, than to envelope it in splints of leather, undressed with oil, first softened in water and allowed to remain on to harden in the exact shape of the joint, when the edges should be rounded and the splints covered with soft wash leather ; a large piece of new jeweller's wool is then to be laid over the patella and upper part of the joints, to prevent too much pressure of the edges ; the splints are afterwards to be applied and fixed by a roller of strong stuff attached to the end of one of the splints, and passed round and round the joint." Our idea, as stated above, was to throw, to a certain extent at least, the weight of the body, during locomotion, on the pelvis, and at the same time to give such general support to the limb as would allow the patient to extend it freely, and bear his weight upon it as fast as returning strength would permit. The lateral support to be such as to protect all the joints from deformity.

The last paragraph of the able author, will we think, apply with equal if not greater force to our apparatus, than it does to his plan.

" Besides giving great support to the joints in walking and standing, resisting the tendency to displacement, and consequently, preventing deformity, the leather (spring) apparatus is particularly serviceable in cases of partial anchylosis of the knee joint, more particularly where adhesive bands had been formed, which are liable to be stretched and even torn, and fresh inflammation to be set up from every little slip in walking, if the joint be not guarded by an efficient apparatus. No strapping or rolling can preserve a joint from the effects of these accidents so well as the leather case, (steel springs.) Being firm, it (they) preserves the joint also from external violence, and lastly, I consider this apparatus very valuable, by supplying an immediate, or rather, we may call it, a prophylactic remedy for inflammatory attacks."

In another case of the same disease, occurring in Mr. M. a carpenter, twenty-four years of age, who had had a severe attack of acute inflammation of the synovial membrane, and other tissues of the right knee joint, producing stiffening of the joint and contraction of the flexor muscles ; I was enabled to restore the limb by means of a single wooden splint and a bandage. The splint was long enough to reach from the tuberosity of the ischium to beyond the heel under the leg, and a bandage, the ordinary muslin roller, was carefully applied, from the ankle to the pelvis, around both leg and splint, binding in this way the angular and deformed limb to a horizontal plane. Frictions with various oleaginous mixtures, and cooling lotions were applied, and the bandage tightened daily, until, in about two weeks, the leg was sufficiently extended to permit him to walk upon the ball of the foot. The frictions with the hands and liniments were continued in the day time, and the splint worn at night, until a complete cure was effected. The high heeled shoe was also strictly forbidden in this case.

In these and many other cases which might be mentioned, in which tenotomy was not resorted to, we are of the opinion that much is due to the fact that they were *recent*, and although, as in the case of Mrs. L., the contraction was very considerable, the leg being almost at right angles with the thigh, yet the contractions of the muscles and the deposits in the vicinity of the joints, not having become old and firm, were the more easily extended and broken up. At the same time these cases may be considered useful, as exhibiting how much may be done by the use of merely mechanical means, combined with appropriate frictions.

## Figures and Tables

**Figure f1:**